# A minimally processed dietary pattern is associated with lower odds of metabolic syndrome among Lebanese adults

**DOI:** 10.1017/S1368980017002130

**Published:** 2017-10-02

**Authors:** Lara Nasreddine, Hani Tamim, Leila Itani, Mona P Nasrallah, Hussain Isma’eel, Nancy F Nakhoul, Joana Abou-Rizk, Farah Naja

**Affiliations:** 1 Department of Nutrition and Food Sciences, Faculty of Agricultural and Food Sciences, American University of Beirut, PO Box 11-0236, Riad El Solh 1107 2020, Beirut, Lebanon; 2 Vascular Medicine Program, American University of Beirut, Beirut, Lebanon; 3 Clinical Research Institute, Faculty of Medicine, American University of Beirut Medical Center, Beirut, Lebanon; 4 Department of Internal Medicine, Faculty of Medicine, American University of Beirut Medical Center, Beirut, Lebanon; 5 Department of Nutrition & Dietetics, Faculty of Health Sciences, Beirut Arab University, Beirut, Lebanon; 6 Department of Internal Medicine, Division of Endocrinology, Faculty of Medicine, American University of Beirut Medical Center, Beirut, Lebanon; 7 Department of Internal Medicine, Division of Cardiology, Faculty of Medicine, American University of Beirut, Beirut, Lebanon

**Keywords:** Food classification system, Ultra-processed foods, Dietary patterns, Metabolic syndrome, Urban adults

## Abstract

**Objective:**

To (i) estimate the consumption of minimally processed, processed and ultra-processed foods in a sample of Lebanese adults; (ii) explore patterns of intakes of these food groups; and (iii) investigate the association of the derived patterns with cardiometabolic risk.

**Design:**

Cross-sectional survey. Data collection included dietary assessment using an FFQ and biochemical, anthropometric and blood pressure measurements. Food items were categorized into twenty-five groups based on the NOVA food classification. The contribution of each food group to total energy intake (TEI) was estimated. Patterns of intakes of these food groups were examined using exploratory factor analysis. Multivariate logistic regression analysis was used to evaluate the associations of derived patterns with cardiometabolic risk factors.

**Setting:**

Greater Beirut area, Lebanon.

**Subjects:**

Adults ≥18 years (*n* 302) with no prior history of chronic diseases.

**Results:**

Of TEI, 36·53 and 27·10 % were contributed by ultra-processed and minimally processed foods, respectively. Two dietary patterns were identified: the ‘ultra-processed’ and the ‘minimally processed/processed’. The ‘ultra-processed’ consisted mainly of fast foods, snacks, meat, nuts, sweets and liquor, while the ‘minimally processed/processed’ consisted mostly of fruits, vegetables, legumes, breads, cheeses, sugar and fats. Participants in the highest quartile of the ‘minimally processed/processed’ pattern had significantly lower odds for metabolic syndrome (OR=0·18, 95 % CI 0·04, 0·77), hyperglycaemia (OR=0·25, 95 % CI 0·07, 0·98) and low HDL cholesterol (OR=0·17, 95 % CI 0·05, 0·60).

**Conclusions:**

The study findings may be used for the development of evidence-based interventions aimed at encouraging the consumption of minimally processed foods.

Food processing is defined as the alteration of food from its whole, natural state to enhance its safety, storage, convenience and palatability^(^ ^1^ ^)^. A number of typologies have been proposed to categorize food products according to their degree of processing^(^ ^1^ ^,^ ^2^ ^)^. One commonly used typology is the NOVA food classification system, which was proposed by the School of Public Health of the University of São Paulo, Brazil^(^ ^3^ ^)^. It assigns foodstuffs to four groups according to the extent and purpose of their industrial processing, including: unprocessed and minimally processed foods (Group 1); processed culinary ingredients (Group 2); processed foods (Group 3); and ultra-processed food and drink (UPF) products (Group 4)^(^ ^3^ ^)^. Compared with unprocessed or minimally processed foods, UPF are typically energy-dense, have a higher glycaemic load and are higher in fat, saturated fat and salt^(^ ^4^ ^,^ ^5^ ^)^. Although few studies have explored the direct association between UPF consumption and health markers, available evidence suggests that higher intakes of UPF are associated with the risk for overweight, obesity^(^ ^6^ ^,^ ^7^ ^)^ and metabolic syndrome (MetS)^(^ ^8^ ^)^, thus highlighting their potential role in modulating the risk of several diet-related non-communicable diseases.

Reports from countries in Europe and Northern and Central America suggest increasing consumption of UPF^(^ ^9^ ^–^ ^13^ ^)^, which are rapidly displacing staple foods and penetrating all segments of the market^(^ ^9^ ^,^ ^11^ ^,^ ^13^ ^–^ ^15^ ^)^. Less is known about the intakes of UPF in low-income countries and those undergoing economic and nutrition transition. Within the context of the increasing globalization of food systems, the theory of the nutrition transition suggests that with economic development, the population’s consumption pattern shifts from minimally processed diets rich in plant-based staple foods to diets high in meat, fats and processed foods^(^ ^16^ ^)^. Recent predictions, therefore, propose that the consumption of UPF will continue to increase in developing countries, raising questions about the implications of this trend on the burden of disease in these countries^(^ ^17^ ^–^ ^19^ ^)^. This may be of direct relevance to the Eastern Mediterranean region, a region that is witnessing rapid rates of development, mechanization and urbanization, accompanied by dietary shifts and alarming increases in the prevalence of non-communicable diseases^(^ ^20^ ^,^ ^21^ ^)^. Lebanon, a small country in the Eastern Mediterranean basin, is no exception to this trend, with non-communicable diseases accounting for over 80 % of the annual deaths nationwide^(^ ^22^ ^)^.

Given the need to characterize the consumption of UPF in countries of the Eastern Mediterranean region and the scarcity of evidence on the link between UPF consumption and health markers, the present study was undertaken to: (i) estimate the consumption levels of minimally processed, processed and ultra-processed foods in a sample of Lebanese adults; (ii) explore the patterns of intakes from minimally processed, processed and ultra-processed foods in the study population; and (iii) investigate the association of the derived patterns with MetS and its components. In addition, the sociodemographic and lifestyle correlates of the identified patterns were examined. In contrast to previous studies that have used household food expenditure or sales data to assess the consumption of processed foods^(^ ^5^ ^,^ ^9^ ^,^ ^11^ ^,^ ^13^ ^,^ ^14^ ^,^ ^23^ ^)^, the current study was based on an individual dietary survey using a culture-specific FFQ. By characterizing the intakes of processed foods in Lebanon and investigating their association with cardiometabolic risk at the population level, the study findings may inform the development of culture-specific interventions that consider food processing and which may contribute to the prevention of non-communicable diseases in Lebanon and other countries in the Eastern Mediterranean region.

## Methods

Data for the present study were drawn from a community-based survey conducted among a representative sample of Lebanese adult participants residing in the Greater Beirut area, which includes the city of Beirut and its suburbs. The survey was carried out between March and May 2014. The survey protocol was approved by the Institutional Review Board of the American University of Beirut and all participants provided written informed consent.

### Study participants

A representative sample of Lebanese adults was selected using a door-to-door sampling strategy and a multistage stratified probability sampling frame. Within this frame, the strata were districts of the Greater Beirut area; within each district, neighbourhoods, then households were selected based on a systematic random sampling^(^ ^24^ ^)^. The numbers of neighbourhoods and households selected within each district were proportional to the total estimated numbers of neighbourhoods and households within the district, respectively. At the household level, when more than one adult respondent was present, information on the month of birth was obtained and the adult respondent with the most recent month of birth was invited to participate, if eligible. The eligibility criteria were age above 18 years, of Lebanese nationality and residing in the Greater Beirut area. Participants were excluded if they were on dialysis, mentally disabled or pregnant. In addition, given that the original survey was designed to examine the population’s exposure to bisphenol A, participants working in plastic or other chemical companies were excluded since they may have been occupationally exposed to bisphenol A. Eligible respondents who agreed to participate in the survey were invited to visit the Department of Nutrition and Food Sciences of the American University of Beirut for data collection. For the present study, the selection of participants from the original survey respondents (*n* 501) was undertaken according to the following criteria: (i) having complete anthropometric, biochemical and dietary data; and (ii) with no known diagnosis of chronic disease or any metabolic abnormality. Accordingly, 302 participants were included in the current analysis. The availability of 302 participants allowed the determination of an OR of 2·15 of MetS in relation to the adherence to a certain dietary pattern, at a power of 80 % and 5 % significance. These calculations were made under the assumption of a 15 % difference in exposure between participants with and without MetS^(^ ^25^ ^)^.

### Data collection

The participants were instructed to visit the Department of Nutrition and Food Sciences after fasting for 10 h and to bring with them all the medications that they were taking at the time of the study. During their visit, the study participants completed a multicomponent questionnaire and an FFQ during a one-on-one interview. In addition, anthropometric measurements, including weight, height and waist circumference, were obtained, and blood samples were drawn by a licensed phlebotomist. Data collection was conducted by trained staff to minimize interviewer and measurement errors. Other quality control measures were applied, including the pre-testing of the study instruments, equipment and data collection procedure, as well as the field monitoring of data collection. The interview and data collection for each participant lasted approximately 1 h.

#### Sociodemographic, anthropometric and biochemical assessment

Using the multicomponent questionnaire, information was obtained about the participants’ age (in years), sex, marital status (married, including those living with a partner, *v.* single, including widowers and divorcees), area of residence (outside Beirut city *v*. Beirut), level of education (elementary to intermediate, secondary or technical, and university or above), monthly income (in Lebanese Liras (LL)), smoking status (non-smoker, including participants who never smoked, *v.* past and current smokers) and physical activity, which was assessed using the short version of the International Physical Activity Questionnaire (IPAQ). Three categories of physical activity (low, moderate and high) were assigned based on MET-min/week, as follows: low, <600; moderate, at least 600; and high, at least 3000^(^ ^26^ ^)^ (where MET=metabolic equivalent of task). Data about past medical history and current health status were also obtained.

Anthropometric measurements were taken using standardized protocols^(^ ^27^ ^)^ and calibrated equipment. Height and body weight were measured using a portable stadiometer (Holtain, Crymych, UK) and a calibrated electronic weighing scale (Seca, Hamburg, Germany), respectively. Participants were weighed to the nearest 0·1 kg in light indoor clothing and with bare feet or stockings. Height was measured without shoes and recorded to the nearest 0·5 cm. All anthropometric measurements were taken twice and the average of the two values was used. BMI was calculated as the ratio of weight (kilograms) to the square of height (metres). Sitting blood pressures, both systolic and diastolic, were obtained twice using a digital sphygmomanometer, at 10 min intervals.

Using the collected blood samples, fasting glucose levels were determined using an enzymatic method (Cobas 6000; Roche, Indianapolis, IN, USA) and levels of TAG and HDL cholesterol (HDL-C) were measured using an enzymatic spectrophotometric technique using a Vitros 350 analyser (Ortho-Clinical Diagnostics, Johnson & Johnson, High Wycombe, UK). MetS was diagnosed based on the harmonized definition from the International Diabetes Federation^(^ ^28^ ^)^, whereby participants were classified as having the MetS if they had three of the five following cardiometabolic risk factors: (i) elevated TAG level (≥150 mg/dl); (ii) low HDL-C level (<40 mg/dl for men, <50 mg/dl for women); (iii) elevated blood pressure (systolic ≥130 mmHg and/or diastolic ≥85 mm Hg); (iv) elevated fasting glucose level (≥100 mg/dl); and (v) elevated waist circumference (≥94 cm for men, ≥80 cm for women).

#### Dietary intake assessment and derivation of dietary patterns

Dietary intake assessment was performed using a culture-specific eighty-item semi-quantitative FFQ which referred to the participants’ dietary intake over the previous year^(^ ^29^ ^)^. To be included in the present study, participants had to have 100 % completion of all questions included in the FFQ. This criterion was met by all participants, given that data collection was interviewer-based and FFQ were checked for completeness after the interview. Participants were asked to record the frequency of their food and beverage consumption per day, per week, per month, per year or never. Participants had the choice to report their intakes in terms of a reference portion size or in grams. The reference portions of the two-dimensional food portion visual^(^ ^30^ ^)^ were used to assist in portion size estimation, in addition to common household measures. The reported frequency of consumption of each food item and beverage was then converted to daily intake. Total energy intake (TEI) was computed using the food composition database of the Nutritionist Pro™ software (Axxya Systems LLC, Stafford, TX, USA). The food items listed in the FFQ were grouped into twenty-five food groups based on the NOVA food classification system. The total consumption for each food group was determined by summing the daily portion intake of each item within the group. Using these twenty-five food groups, exploratory factor analysis was implemented to identify patterns of dietary intake. The rotated factor loadings matrix was extracted (Varimax rotation). The derived dietary patterns were labelled based on food groups having a rotated factor loading greater than |0·4|. The factor scores were calculated using multiple regression and were categorized, based on the total sample distribution, into first, second, third and fourth quartiles corresponding to low (first quartile) and medium/high adherence levels (second, third and fourth quartiles).

### Statistical analysis

Frequencies, means and standard deviations were used to describe the various sociodemographic, lifestyle, anthropometric and clinical characteristics of the overall study population and by MetS status. Categorical and continuous variables were compared between participants with and without MetS using *χ* ^2^ and *t* tests, respectively. For the twenty-five food groups considered, percentage contribution to TEI was calculated as mean and standard deviation. Multiple logistic regression analyses were used to examine the associations of dietary patterns with MetS and each of its components. For each pattern, six independent regression models were built whereby the dependent variables were MetS and its five components and the independent variable was the adherence to the pattern (low *v*. medium/high). The latter was grouped as such to examine the odds of MetS among participants with low adherence to a certain pattern (belonging to the first quartile) compared with those with higher adherence (belonging to the second, third and fourth quartiles) to this pattern. Analyses for model 1 were adjusted for all sociodemographic and lifestyle characteristics considered in the study (age, sex, marital status, area of residence, level of education, income, smoking status and physical activity) as well as TEI. Model 2 included BMI in addition to all the variables included in model 1.

Multiple logistic regression analyses were also used to assess the sociodemographic and lifestyle correlates of the dietary patterns, using the sociodemographic and lifestyle characteristics as independent variables and adherence to each of the identified patterns as the dependent variables (low (first quartile) *v*. medium/high (second, third and fourth quartiles)). *P*<0·05 indicated statistical significance. The statistical software package IBM SPSS Statistics for Windows version 22·0 was used for data cleaning, management and analysis.

## Results

The sociodemographic, lifestyle and anthropometric characteristics, and cardiometabolic risk factors of the study population are presented in Table 1. Overall, the mean age of the participants was 39·35 (sd 13·84) years, with a higher proportion of female than male participants (61·3 *v*. 38·7 %). Three out of four participants (76·6 %) were living in the city of Beirut and 15·0 % had a university level of education. Only 12·3 % of the study participants had a monthly income level higher than 3 million LL (approximately $US 2000). Among lifestyle factors, including smoking and physical activity, 71·5 % were current smokers and 44·4 % had a low level of physical activity. The comparison of participants with and without MetS showed that those with MetS were significantly older (43·41 (sd 14·69) *v*. 37·25 (sd 12·89) years) and a higher proportion was male (51·4 *v*. 31·8 %). Also, a higher proportion of participants with lower education levels (elementary to intermediate education) was noted among participants with MetS compared with those without MetS (66·4 *v*. 52·8 %). Higher prevalence rates of elevated fasting blood glucose and TAG levels, low HDL-C level, elevated blood pressure, elevated waist circumference and obesity were noted among participants with MetS than among those without (Table 1).

**Table 1:** Sociodemographic, lifestyle and anthropometric characteristics and cardiometabolic risk factors of the study population of Lebanese adults (*n* 302), Greater Beirut area, March–May 2014

	All participants (*n* 302)		With metabolic syndrome* (*n* 195)		Without metabolic syndrome (*n* 107)			
Variables	Mean or *n*	sd or %	Mean or *n*	sd or %	Mean or *n*	sd or %	Significance† (*χ* ^2^)	*P* value
Age (years)	39·35	13·84	43·41	14·69	37·25	12·89		1·94×10^−4^
Sex								
Male	117	38·7	55	51·4	62	31·8	11·190	0·001
Female	185	61·3	52	48·6	133	68·2		
Marital status								
Single	109	36·1	38	35·5	71	36·4	0·024	0·877
Married	193	63·9	69	64·5	124	63·6		
Area of residence								
Outside the city of Beirut	68	23·4	21	20·0	47	25·4	1·090	0·296
Beirut city	222	76·6	84	80·0	138	74·6		
Level of education								
Elementary to intermediate	173	57·7	71	66·4	102	52·8	8·536	0·014
Secondary or technical	82	27·3	28	26·2	54	28·0		
University	45	15·0	8	7·5	37	19·2		
Income (LL/month)								
<1·5 million	184	66·4	74	71·8	110	63·2	2·231	0·328
1·5–3 million	59	21·3	19	18·4	40	23·0		
>3 million	34	12·3	10	9·7	24	13·8		
Smoking status								
Non-smoker	86	28·5	31	29·0	55	28·2	0·020	0·888
Current smoker	216	71·5	76	71·0	140	64·8		
Physical activity level								
Low intensity	134	44·4	52	48·6	82	42·1	2·555	0·279
Moderate intensity	96	31·8	35	32·7	61	31·3		
High intensity	72	23·8	20	18·7	52	26·7		
Fasting blood glucose (mg/dl)								
Normal	194	64·9	32	30·8	162	83·1	81·448	1·799×10^−19^
Elevated (≥100 mg/dl)	105	35·1	72	69·2	33	16·9		
TAG level (mg/dl)								
Normal	220	73·6	48	46·2	172	88·2	61·695	4·011×10^−15^
Elevated (≥150 mg/dl)	79	26·4	56	53·8	23	11·8		
HDL-C level (mg/dl)								
Normal	183	61·6	37	34·6	146	76·8	51·695	6·482×10^−13^
Low (males <40 mg/dl, females <50 mg/dl)	114	38·4	70	65·4	44	23·2		
Blood pressure								
No	217	71·9	45	42·1	172	88·2	72·757	1·467×10^−17^
Elevated (systolic ≥130 mmHg and/or diastolic ≥85 mmHg)	85	28·1	62	57·9	23	11·8		
Waist circumference (cm)								
Normal	99	32·8	9	8·4	90	46·2	44·664	2·339×10^−11^
Elevated (males ≥94 cm, females ≥80 cm)	203	67·2	98	91·6	105	53·8		
BMI (kg/m^2^)								
Not obese (BMI<30 kg/m^2^)	210	69·5	51	47·7	159	81·5	37·426	9·494×10^−10^
Obese (BMI≥30 kg/m^2^)	92	30·5	56	52·3	36	18·5		

LL, Lebanese Lira; HDL-C, HDL cholesterol. Data are presented as mean and sd for continuous variable (age) or as *n* and % for categorical variables.

* Metabolic syndrome was defined according to Alberti *et al.* ^(^ ^28^ ^)^.

† Significance was obtained using the *t* test and the *χ* ^2^ test for continuous and categorical variables, respectively.

Table 2 presents the dietary intakes of various food groups in the study population, based on the NOVA food classifications of unprocessed and minimally processed foods, processed culinary ingredients, processed foods and UPF. Listed within the unprocessed and minimally processed foods category were the following food groups: ‘water’, ‘whole and low-fat milk and dairy products’, ‘fruits, fruit juices (fresh) and vegetable juices’, ‘vegetables and legumes’, ‘meat (fish, red meat and poultry)’, ‘eggs’, ‘refined/whole grains and pasta’, ‘Turkish coffee’ and ‘olives’. The percentage contribution to TEI from this category was 27·10 %, with ‘fruits, fruit juices (fresh) and vegetable juices’ having the highest percentage (5·98 %). The processed culinary ingredients category included two food groups: ‘olive oil, vegetable oil, butter, ghee and tahini’ and ‘sugar, honey’ and molasses’; this category had a 12·25 % contribution to TEI. The processed foods category contributed 23·83 % to TEI and included ‘wine and beer’, ‘nuts and seeds (roasted and salted)’, ‘processed cheeses’, ‘canned foods (vegetables, legumes and fish)’ and ‘breads’. Within this category, ‘breads’ had the highest contribution to TEI (18·21 %). The UPF category included ‘canned red and luncheon meats’, ‘pre-fried French fries’, ‘liquor’, ‘condiments (mayonnaise, ketchup and mustard)’, ‘fast-food sandwiches and pizzas’, ‘chips and salty snacks (including tortillas and pretzels)’, ‘sweets and sweetened beverages’ and ‘sausages (including canned)’. The contribution of this category to TEI was the highest, amounting to 36·53 %, with the highest percentage coming from ‘sweets and sweetened beverages’ (15·67 %; Table 2). Comparing participants with MetS and those without, significantly higher contributions to TEI were noted for the ‘breads’ food group (20·40 *v*. 17·21 %, respectively) and processed foods category (25·83 *v*. 22·96 %, respectively). Analysis of the daily portion intake of the various food groups for all participants and by MetS was also conducted; the results are shown in the Appendix.

**Table 2:** Percentage contribution to total energy intake of various food groups in the study population of Lebanese adults (*n* 302), Greater Beirut area, March–May 2014

		All participants (*n* 302)		With metabolic syndrome (*n* 107)		Without metabolic syndrome (*n* 195)		
Type of processing	Type of food	Mean	sd	Mean	sd	Mean	sd	*P* value*
Minimally processed foods	Water	0	0	0	0	0	0	
	Whole milk and dairy products	3·05	3·09	2·63	3·13	3·27	3·07	0·088
	Low-fat milk and dairy products	2·16	3·69	2·26	4·97	2·12	2·79	0·757
	Fruits, fruit juices (fresh) and vegetable juices	5·98	5·37	5·49	4·27	6·29	5·91	0·218
	Vegetables and legumes	3·89	2·86	3·51	2·32	4·08	3·07	0·095
	Meat (fish, red meat and poultry)	5·56	6·10	4·83	4·15	5·97	6·95	0·122
	Eggs	1·09	1·28	1·25	1·48	1·01	1·15	0·131
	Refined/whole grains and pasta	4·88	4·14	5·01	4·99	4·79	3·63	0·658
	Turkish coffee	0·03	0·08	0·03	0·06	0·03	0·09	0·739
	Olives	0·46	0·76	0·44	0·62	0·48	0·84	0·692
	Total	27·10	11·83	25·45	11·28	28·04	12·26	0·070
Processed culinary ingredients	Olive oil, vegetable oil, butter, ghee and tahini	10·42	8·38	10·85	8·69	10·17	8·21	0·500
	Sugar, honey and molasses	1·83	3·65	2·30	5·36	1·59	2·23	0·110
	Total	12·25	9·13	13·14	9·83	11·76	8·72	0·208
Processed foods	Wine and beer	0·35	1·40	0·31	1·04	0·37	1·58	0·714
	Nuts and seeds (roasted and salted)	3·07	4·55	2·84	4·84	3·23	4·42	0·471
	Processed cheeses	1·32	1·89	1·38	1·53	1·29	2·06	0·582
	Canned food (vegetables, legumes and fish)	0·88	1·14	0·90	1·36	0·86	1·01	0·768
	Breads	18·21	11·00	20·40	11·84	17·21	10·32	0·015
	Total	23·83	11·69	25·83	12·07	22·96	11·28	0·040
Ultra-processed foods	Canned red and luncheon meats	0·36	0·67	0·28	0·46	0·40	0·76	0·133
	Pre-fried French fries	6·91	6·73	6·34	5·92	7·29	7·16	0·242
	Liquor	0·76	3·48	0·57	1·95	0·88	4·10	0·469
	Condiments (mayonnaise, ketchup and mustard)	0·50	0·78	0·39	0·56	0·56	0·87	0·057
	Fast-food sandwiches and pizzas	8·8	6·37	8·57	6·03	8·79	6·51	0·769
	Chips and salty snacks (including tortillas and pretzels)	3·05	5·10	2·29	3·85	3·37	5·57	0·078
	Sweets and sweetened beverages	15·67	11·13	16·67	12·07	15·02	10·51	0·215
	Sausages (including canned)	0·49	1·33	0·34	0·57	0·56	1·61	0·163
	Total	36·53	16·51	35·45	15·97	36·87	16·77	0·473

* Significance was derived from the independent *t* test.

Factor analysis revealed two main dietary patterns: ‘ultra-processed’ and ‘minimally processed/processed’, which explained 11·79 and 10·65 % of the dietary intake variance, respectively (Table 3). Factor loadings showed that the ‘ultra-processed’ pattern was characterized by high intakes of: pre-fried French fries; condiments; fast-food sandwiches and pizzas; chips and salty snacks; sausages; nuts and seeds; sweets and sweetened beverages; canned red and luncheon meats; liquor; low-fat milk and dairy products; and meat (fish, red meat and poultry). The ‘minimally processed/processed’ pattern consisted mostly of: fruits, fruit juices (fresh) and vegetable juices; vegetables and legumes; canned food (fruits, vegetables, legumes and fish); refined and whole grains; processed cheeses; whole milk and dairy products; sugar, honey and molasses; breads; and olive oil, vegetable oil, butter, ghee and tahini. The factor loading matrix of these patterns is shown in Table 3.

**Table 3:** Factor loading matrix* of the dietary patterns, derived using factor analysis, among the study population of Lebanese adults (*n* 302), Greater Beirut area, March–May 2014

	‘Ultra-processed’	‘Minimally processed/processed’
Pre-fried French fries	0·72	–
Condiments (mayonnaise, ketchup and mustard)	0·65	–
Fast-food sandwiches and pizzas	0·52	–
Chips and salty snacks (including tortillas and pretzels)	0·48	−0·21
Sausages (including canned)	0·44	–
Nuts and seeds (roasted and salted)	0·44	0·20
Sweets and sweetened beverages	0·43	0·34
Canned red and luncheon meats	0·41	0·24
Liquor	0·40	–
Low fat-milk and dairy products	0·31	–
Meat (fish, red meat and poultry)	0·30	0·25
Eggs	0·27	–
Fruits, fruit juices (fresh) and vegetable juices	–	0·59
Vegetables and legumes	–	0·58
Canned food (fruits, vegetables, legumes and fish)	–	0·55
Refined and whole grains	–	0·53
Processed cheeses	–	0·50
Whole milk and dairy products	0·25	0·37
Sugar, honey and molasses	–	0·35
Breads	0·26	0·33
Wine and beer	0·32	0·32
Olive oil, vegetable oil, butter, ghee and tahini	–	0·30
Olives	–	0·25
Water	–	0·25
Turkish coffee	–	0·18
Variance explained (%)	11·79	10·65

* Loadings <|0·1| were removed for simplicity.

The associations of the derived intake patterns with MetS and its components were examined using multiple logistic regression, with MetS and its five components as dependent variables and adherence to the pattern (low *v*. medium/high) as the independent variable (Table 4). For the ‘ultra-processed’ pattern, no association was observed with MetS or any of its components. For the ‘minimally processed/processed’ pattern, a medium/high adherence was associated with 75 % lower odds of having hyperglycaemia (OR=0·25; 95 % CI 0·07, 0·98) and approximately 80 % lower odds of having low HDL-C level (OR=0·17; 95 % CI 0·05, 0·60) and MetS (OR=0·18; 95 % CI 0·04, 0·77). When BMI was added to the multiple logistic regression models, only the association of the ‘minimally processed/processed’ pattern with hyperglycaemia was attenuated by 2 % and became non-significant (Table 4).

**Table 4:** Multiple logistic regression of the associations among the derived dietary patterns and the odds of metabolic syndrome and its components in the study population of Lebanese adults (*n* 302), Greater Beirut area, March–May 2014

	Components of metabolic syndrome											
	High blood glucose		High TAG		Low HDL-C		Elevated blood pressure		Elevated waist circumference		Metabolic syndrome	
	OR	95 % CI	OR	95 % CI	OR	95 % CI	OR	95 % CI	OR	95 % CI	OR	95 % CI
‘Ultra-processed’												
Low adherence	1·00	Ref.	1·00	Ref.	1·00	Ref.	1·00	Ref.	1·00	Ref.	1·00	Ref.
Medium/high adherence (model 1)*	0·57	0·17, 1·93	0·89	0·25, 3·20	2·29	0·67, 7·85	3·17	0·64, 15·64	0·28	0·02, 3·35	1·28	0·32, 5·16
Medium/high adherence (model 2)†	0·52	0·15, 1·83	1·08	0·28, 4·11	1·82	0·52, 6·42	3·10	0·58, 16·66	0·20	0·01, 3·88	1·11	0·26, 4·65
‘Minimally processed/processed’												
Low adherence	1·00	Ref.	1·00	Ref.	1·00	Ref.	1·00	Ref.	1·00	Ref.	1·00	Ref.
Medium/high adherence (model 1)*	**0**·**25**	**0**·**07**, **0**·**98**	0·41	0·11, 1·50	**0**·**17**	**0**·**05**, **0**·**60**	0·70	0·14, 3·54	1·48	0·60, 3·66	**0**·**18**	**0**·**04**, **0**·**77**
Medium/high adherence (model 2)†	0·27	0·07, 1·04	0·44	0·12, 1·60	**0**·**12**	**0**·**03**, **0**·**47**	0·67	0·13, 3·46	1·87	0·53, 6·60	**0**·**21**	**0**·**05**, **0**·**87**

HDL-C, HDL cholesterol; low adherence, first quartile of pattern score; medium/high adherence, second, third and fourth quartiles of the pattern score; Ref., reference category.

* OR were adjusted for age, gender, marital status, area of residence, level of education, income, smoking status, physical activity and total energy intake. Significant results are shown in bold.

† OR were adjusted to BMI in addition to all the variables adjusted for in model 1. Significant results are shown in bold.

To examine the sociodemographic and lifestyle correlates of the dietary patterns, two multiple logistic regression models were built, with an outcome of medium/high *v*. low adherence to the patterns (Table 5). The results showed that age was significantly positively associated with adherence to the ‘minimally processed/processed’ pattern (OR=1·04; 95 % CI 1·01, 1·07) and significantly negatively associated with adherence to the ‘ultra-processed’ pattern (OR=0·93; 95 % CI 0·90, 0·96). Education level was also significantly associated with both patterns, with a university education level negatively associated with adherence to the ‘ultra-processed’ pattern (OR=0·26; 95 % CI 0·07, 0·87) and positively associated with adherence to the ‘minimally processed/processed’ pattern (OR=4·05; 95 % CI 1·20, 13·67). Furthermore, compared with non-smokers, current smokers had 50 % lower odds of adhering to the ‘minimally processed/processed’ pattern (OR=0·47; 95 % CI 0·15, 0·84; Table 5).

**Table 5:** Sociodemographic, lifestyle and anthropometric correlates of the identified dietary patterns, assessed using multiple logistic regression*, in the study population of Lebanese adults (*n* 302), Greater Beirut area, March–May 2014

	Adherence to dietary pattern (medium and high adherence *v.* low)			
	‘Ultra-processed’		‘Minimally processed/processed’	
	OR	95 % CI	OR	95 % CI
Age	**0**·**93**	**0**·**90**, **0**·**96**	**1**·**04**	**1**·**01**, **1**·**07**
Sex				
Female	0·78	0·31, 1·94	0·66	0·29, 1·51
Marital status				
Married	0·92	0·39, 2·13	0·76	0·36, 1·58
Area of residence				
Beirut	0·46	0·20, 1·04	1·30	0·58, 2·89
Level of education				
Secondary or technical	0·78	0·31, 1·99	1·84	0·81, 4·17
University	**0**·**26**	**0**·**07**, **0**·**87**	**4**·**05**	**1**·**20**, **13**·**67**
Income (LL/month)				
1·5–3 million	0·63	0·25, 1·55	0·88	0·38, 2·04
>3 million	1·94	0·47, 8·02	1·12	0·34, 3·71
Smoking				
Current smoker	0·90	0·41, 1·94	**0**·**47**	**0**·**15**, **0**·**84**
Physical activity level				
Moderate intensity	0·92	0·41, 2·09	1·68	0·77, 3·70
High intensity	0·62	0·25, 1·51	0·64	0·30, 1·34

LL, Lebanese Lira.

* OR and 95 % CI were derived from multiple regression models. For each pattern, a logistic regression model was built with all the variables listed in the table as independent variables while the dependent variable was low adherence (first quartile) *v.* medium/high adherence (second, third and fourth quartiles) to the pattern. Significant results are shown in bold.

A summary of selected findings described in this section was presented elsewhere^(^ ^31^ ^)^.

## Discussion

Using a food classification system that takes account of the nature, extent and purpose of food processing^(^ ^3^ ^)^, the present study characterized the consumption of minimally processed, processed and ultra-processed foods in a sample of Lebanese adults and showed that UPF provided one-third of the daily energy intake of adults in Lebanon. The study also explored the overall patterns of intakes of these food groups within the population. It identified two main dietary patterns, the ‘ultra-processed’ and ‘minimally processed/processed’, and showed that higher adherence to the ‘minimally processed/processed’ pattern was significantly associated with lower odds for MetS, hyperglycaemia and low HDL-C level.

When compared with estimates reported from other countries using the same food classification system, the intake of UPF in Lebanon (36·53 % of TEI) exceeded that reported in another developing country, Brazil (29·6 %)^(^ ^11^ ^)^, while being lower than estimates described in developed countries such as the USA (57·9 %), Canada (55 %) and Norway (49 %)^(^ ^9^ ^,^ ^14^ ^,^ ^15^ ^)^. These findings are in agreement with previous reports concluding that the consumption of convenient, ultra-processed and ready-to-eat foods is currently the greatest in developed, high-income countries, while also predicting the gradual establishment of similar consumption patterns in developing countries given the ongoing nutrition transition, increased food systems globalization and the adoption of aggressive marketing strategies for this type of food^(^ ^16^ ^)^. Compared with data reported in other countries, the consumption of the processed foods category was the highest in Lebanon (23·83 *v*. 2·5–9·4 % of TEI)^(^ ^1^ ^,^ ^9^ ^,^ ^11^ ^,^ ^13^ ^–^ ^15^ ^,^ ^23^ ^)^. This may be explained by the fact that in the present study, bread, which is considered a main staple food in Lebanon, was included in the processed food category, and not in the UPF group, given that Lebanese breads do not contain additives and are purchased fresh from bakeries rather than as pre-packaged food products^(^ ^32^ ^)^. The study findings also show that the consumption of unprocessed and minimally processed foods and processed culinary ingredients combined provided approximately 40 % of TEI in Lebanon, a value that falls within the range reported in several developed countries (32·5–55 %)^(^ ^1^ ^,^ ^9^ ^,^ ^11^ ^,^ ^13^ ^–^ ^15^ ^,^ ^23^ ^)^. This suggests that, in line with the theory of nutrition transition and globalization of the food system, home-based food preparations and cooking are becoming less dominant in the dietary habits of Lebanese adults.

It is important to note that the comparison of data stemming from the present study with those reported from other countries should be interpreted with caution, since in many instances the studies may have differed in dietary assessment methodologies. While the present study was based on an individual dietary survey, most of the other studies were based on household expenditures, retail-based sales or per capita food availability data^(^ ^9^ ^,^ ^11^ ^,^ ^13^ ^,^ ^14^ ^)^, which may not reflect actual food consumption at the individual level^(^ ^33^ ^)^.

Beyond the characterization of the consumption of unprocessed, processed and ultra-processed foods in terms of contribution to energy intake, the current study explored overall patterns of intakes of these foods and their associations with cardiometabolic risk. This approach of examining overall dietary patterns is receiving increasing attention in nutrition research as it may better reflect the intakes of foods as normally consumed. This approach may also overcome the limitations of the traditional methods of examining single foods or food groups, thus allowing investigation of the joint effects of multiple dietary components on disease risk^(^ ^34^ ^,^ ^35^ ^)^. The study findings revealed two main dietary patterns, the ‘ultra-processed’ and the ‘minimally processed/processed’ patterns, which together explained almost 22 % of the dietary intake variance. As its name implies, the ‘ultra-processed’ pattern was characterized by high intakes of convenient, ready-to-eat food items, while also including meat (fish, red meat and poultry), roasted nuts and seeds, and low-fat dairy products. The ‘minimally processed/processed’ pattern included processed foods such as breads and canned food, but was mainly characterized by high intakes of minimally processed, plant-based foods such as fruits, vegetables, legumes and grains. As such, the ‘minimally processed/processed’ pattern shared many of the characteristics of the traditional Lebanese dietary pattern, which was previously described as a variant of the Mediterranean diet^(^ ^36^ ^)^.

The study findings did not indicate a direct association between the ‘ultra-processed’ pattern and MetS or any of its components. While other studies have examined the association of cardiometabolic risk with the intake of UPF as an *a priori* set food group^(^ ^7^ ^,^ ^8^ ^,^ ^17^ ^)^, the method adopted in the present study allowed for the examination of food group intake patterns as normally consumed by the population. The fact that the ‘ultra-processed’ pattern included nuts and seeds, fish and low-fat dairy products in addition to UPF may have diluted the association between UPF and metabolic abnormalities. Interestingly, the present results showed that a higher adherence to the ‘minimally processed/processed’ dietary pattern was associated with lower odds of having hyperglycaemia, low HDL-C level and MetS. In contrast to UPF, minimally processed foods tend to retain their nutritional properties and characteristics. As such, the beneficial combinations of phytochemicals, antioxidants, fibre and monounsaturated fats brought by legumes, fruits, vegetables, grains and olives/olive oil, which are among the main characteristics of the ‘minimally processed/processed’ pattern, may work in concert to decrease oxidative stress, temper the inflammatory response, enhance fat oxidation, buffer lipid and insulin fluctuations and peaks, improve insulin sensitivity and decrease cardiometabolic risk^(^ ^37^ ^–^ ^41^ ^)^. The observed protective association of a diet rich in minimally processed foods and low in UPF against MetS has public health implications given the high prevalence of cardiometabolic risk factors among Lebanese adults. In agreement with the results of the present study, a previous national study showed that 34·6 % of healthy Lebanese adults had MetS^(^ ^42^ ^)^.

The current study also examined the sociodemographic and lifestyle correlates of the identified dietary patterns. The results showed that older age and higher educational levels were associated with increased adherence to the ‘minimally processed/processed’ dietary pattern. These findings agree with those reported by previous studies conducted in Lebanon and elsewhere, where adherence to ‘traditional’ dietary patterns, which are typically characterized by higher intakes of minimally processed foods and lower consumption of UPF, tended to increase with age and with level of education^(^ ^43^ ^–^ ^47^ ^)^. Our study also showed that, compared with non-smokers, current smokers had significantly lower adherence to the ‘minimally processed/processed’ pattern. These findings may be viewed as a recurrent manifestation of the well-documented clustering of behavioural risk factors, including smoking and unbalanced diet^(^ ^48^ ^)^, a clustering that is increasingly common in countries undergoing the nutrition transition, including Lebanon and other countries in the Eastern Mediterranean region^(^ ^20^ ^)^.

The results of the present study should be considered in light of the following limitations. First, the findings of the study cannot infer causality given its cross-sectional design. Hence, the significant study findings are reflective of associational relationship between exposure and outcome and cannot be used to show a cause–effect relationship. However, to decrease possible reverse causation, participants who reported the diagnosis of a chronic disease or metabolic abnormalities that may have affected their dietary habits were excluded from the study. Second, examining the associations between various dietary patterns with MetS and its components in the current study necessitated multiple statistical testing. Therefore, the possibility of a type I error or detection of false positive results cannot be ruled out. Third, the study was based on the use of an FFQ for the assessment of dietary intake, which may be limited by measurement errors, reliance on memory and the number of food items included in the food list^(^ ^49^ ^)^. However, in the present study, the level of detail of the food list allowed the disaggregation of several food items according to their level of processing. For instance, the questionnaire allowed respondents to distinguish between the consumption of processed meat *v*. unprocessed meat, canned vegetables *v*. fresh vegetables, canned fish *v*. fresh fish, etc. Furthermore, for some foods in the food list, additional probing questions were used to obtain additional information about the foods usually consumed by the individual. Similarly, and to attenuate the restriction imposed by the fixed food list of the FFQ, a question about ‘other foods usually consumed’ was asked at the end of the interview. It is important to note that although the FFQ used in the present study was not validated in our study population, it was previously used for the assessment of dietary patterns and their relationship with obesity and MetS among Lebanese adults and has yielded plausible findings^(^ ^42^ ^,^ ^50^ ^)^. Despite the potential limitations of the FFQ method, it has been shown to be one of the most suitable dietary assessment tools in large epidemiological studies as it provides information on the participants’ habitual diet over longer periods of time^(^ ^51^ ^)^. The FFQ used in the present study was administered by trained nutritionists rather than being self-administered. This approach provides several advantages, as self-administration of the FFQ requires a literate population, and may result in inconsistent interpretations of the food list and lower response and completion rates, each of which may jeopardize the validity of the data^(^ ^51^ ^)^. However, as observed in most questionnaire-based surveys, the interview-based approach may incur a social desirability bias, whereby the survey participants may respond in a manner that they perceive as acceptable or favourable to the interviewer^(^ ^52^ ^)^. In our study, the fieldworkers who conducted data collection had received extensive training to reduce judgemental verbal and non-verbal communication in order to minimize any social desirability bias. The FFQ and the IPAQ used in the present study were not validated in our population; however, these tools were previously used in dietary and physical assessment studies among Lebanese adults and the findings of these studies yielded plausible associations with obesity, MetS, diabetes and metabolic health^(^ ^29^ ^,^ ^42^ ^,^ ^50^ ^,^ ^53^ ^–^ ^59^ ^)^. Finally, the present study was confined to the urban setting of the Greater Beirut area; hence, findings pertinent to the consumption levels of foods may not be representative of less urban settings in the country. The choice of Beirut can be explained by the fact that it comprises 40 % of the Lebanese population and is usually considered a melting pot of the country.

## Conclusion

In conclusion, to our knowledge, the current study is the first to characterize, based on a food classification system, the consumption of unprocessed, processed and ultra-processed foods in the Eastern Mediterranean region, where data on the intakes of these food categories are completely lacking. The results showed that one-third of the energy intake of adults in Lebanon is derived from UPF, thus highlighting the impact that the globalization of food systems and aggressive UPF marketing strategies may have on dietary habits within the population. The study also identified two main dietary patterns in the population, the ‘ultra-processed’ and the ‘minimally processed/processed’, and documented an inverse association between MetS and adherence to the ‘minimally processed/processed’ pattern. These findings may be used for the development of evidence-based interventions and public health strategies aiming to encourage the consumption of minimally processed foods in Lebanon and the region.

## Appendix

***Daily portion intake of various food groups in the study population of Lebanese adults (*** **n** ***302), Greater Beirut area, March–May 2014***

[Table tab6]

**. tab6:** .

		All participants (*n* 302)		With metabolic syndrome (*n* 107)		Without metabolic syndrome (*n* 195)		
Type of processing	Type of food	Mean	sd	Mean	sd	Mean	sd	*P* value
Minimally processed foods	Water	5·09	4·58	5·22	4·81	5·00	4·45	0·659
	Whole milk and dairy products	1·01	1·23	0·90	1·22	1·07	1·24	0·250
	Low-fat milk and dairy products	0·58	0·85	0·56	0·79	0·60	0·89	0·647
	Fruits, fruit juices (fresh) and vegetable juices	2·58	2·16	2·59	1·93	2·60	2·29	0·957
	Vegetables and legumes	3·16	2·50	3·18	2·57	3·17	2·48	0·965
	Meat (fish, red meat and poultry)	1·60	2·55	1·42	1·71	1·71	2·92	0·350
	Eggs	0·43	0·56	0·49	0·63	0·40	0·52	0·205
	Refined/whole grains and pasta	2·00	2·02	2·04	2·13	1·97	1·81	0·779
	Turkish coffee	1·71	3·95	1·80	3·62	1·69	4·14	0·811
	Olives	0·38	0·64	0·39	0·65	0·38	0·63	0·814
Processed culinary ingredients	Olive oil, vegetable oil, butter, ghee and tahini	9·27	8·5	10·16	9·47	8·84	7·96	0·197
	Sugar, honey and molasses	1·31	5·91	2·07	9·84	0·90	1·17	0·103
Processed foods	Wine and beer	0·13	0·52	0·11	0·34	0·14	0·59	0·565
	Nuts and seeds (roasted and salted)	0·43	0·71	0·43	0·77	0·44	0·68	0·900
	Processed cheeses	0·25	0·39	0·28	0·38	0·24	0·39	0·294
	Canned food (vegetables, legumes and fish)	0·37	0·57	0·36	0·54	0·37	0·59	0·950
	Breads	9·28	7·15	10·30	7·09	8·82	7·14	0·086
Ultra-processed foods	Canned red and luncheon meats	0·07	0·15	0·06	0·12	0·08	0·16	0·465
	Pre-fried French fries	1·18	1·44	1·05	1·05	1·26	1·63	0·237
	Liquor	0·52	2·76	0·34	1·27	0·63	3·33	0·382
	Condiments (mayonnaise, ketchup and mustard)	0·84	1·71	0·60	0·89	0·98	2·02	0·069
	Fast-food sandwiches and pizzas	2·90	2·95	2·83	2·47	2·93	3·21	0·760
	Chips and salty snacks (including tortillas and pretzels)	1·02	1·88	0·81	1·37	1·13	2·10	0·160
	Sweets and sweetened beverages	6·03	6·13	6·54	7·40	5·73	5·34	0·279
	Sausages (including canned)	0·10	0·46	0·05	0·09	0·13	0·57	0·167
